# Variability in spring phytoplankton blooms associated with ice retreat timing in the Pacific Arctic from 2003–2019

**DOI:** 10.1371/journal.pone.0261418

**Published:** 2021-12-16

**Authors:** Hisatomo Waga, Hajo Eicken, Toru Hirawake, Yasushi Fukamachi

**Affiliations:** 1 International Arctic Research Center, University of Alaska Fairbanks, Fairbanks, Alaska, United States of America; 2 Arctic Research Center, Hokkaido University, Sapporo, Hokkaido, Japan; 3 Faculty of Fisheries Sciences, Hokkaido University, Hakodate, Hokkaido, Japan; Universidade de Lisboa Instituto Superior Tecnico, PORTUGAL

## Abstract

The Arctic is experiencing rapid changes in sea-ice seasonality and extent, with significant consequences for primary production. With the importance of accurate monitoring of spring phytoplankton dynamics in a changing Arctic, this study further examines the previously established critical relationship between spring phytoplankton bloom types and timing of the sea-ice retreat for broader temporal and spatial coverages, with a particular focus on the Pacific Arctic for 2003–2019. To this end, time-series of satellite-retrieved phytoplankton biomass were modeled using a parametric Gaussian function, as an effective approach to capture the development and decay of phytoplankton blooms. Our sensitivity analysis demonstrated accurate estimates of timing and presence/absence of peaks in phytoplankton biomass even with some missing values, suggesting the parametric Gaussian function is a powerful tool for capturing the development and decay of phytoplankton blooms. Based on the timing and presence/absence of a peak in phytoplankton biomass and following the classification developed by the previous exploratory work, spring bloom types are classified into three groups (under-ice blooms, probable under-ice blooms, and marginal ice zone blooms). Our results showed that the proportion of under-ice blooms was higher in the Chukchi Sea than in the Bering Sea. The probable under-ice blooms registered as the dominant bloom types in a wide area of the Pacific Arctic, whereas the marginal ice zone bloom was a relatively minor bloom type across the Pacific Arctic. Associated with a shift of sea-ice retreat timing toward earlier dates, we confirmed previous findings from the Chukchi Sea of recent shifts in phytoplankton bloom types from under-ice blooms to marginal ice zone blooms and demonstrated that this pattern holds for the broader Pacific Arctic sector for the time period 2003–2019. Overall, the present study provided additional evidence of the changing sea-ice retreat timing that can drive variations in phytoplankton bloom dynamics, which contributes to addressing the detection and consistent monitoring of the biophysical responses to the changing environments in the Pacific Arctic.

## Introduction

Phytoplankton blooms in the Arctic have typically been defined as enhanced growth of phytoplankton populations that occur along the seasonally retreating ice edge, with water column stratification from sea-ice melt providing sufficient light for photosynthesis [1]. Such marginal ice zone (MIZ) blooms are viewed as ubiquitous features throughout the Arctic [2]. In contrast, phytoplankton production beneath sea ice has been deemed negligible because of strong light attenuation by snow and sea-ice cover. However, the discovery in 2011 of a phytoplankton bloom beneath sea ice (under-ice bloom; UI bloom) calls for a revision of our understanding of phytoplankton phenology in the Arctic and primary production beneath sea ice [3]. Further field surveys revealed under-ice blooms across the Arctic, indicating that under-ice blooms are a ubiquitous phenomenon in the Arctic [4–6].

Arctic annual surface air temperatures in 2014–2018 were higher than in any prior years since observational records began in 1900 [7]. The most conspicuous sign associated with this warming is a drastic loss of summer sea-ice extent [8], as well as a replacement of thicker multi-year ice by thinner first-year ice [9] with a significantly higher melt pond fraction [10]. Because melt-pond covered ice transmits three to ten times more light than bare ice of the same thickness [11], the presence of more first-year ice with a higher melt pond fraction leads to an increase in the illumination of the water column beneath the sea ice [12]. These key changes in sea-ice type drive an increase in irradiance in the water column beneath sea ice, resulting in favorable under-ice light fields for the development of under-ice blooms [5].

**Fig 1:**
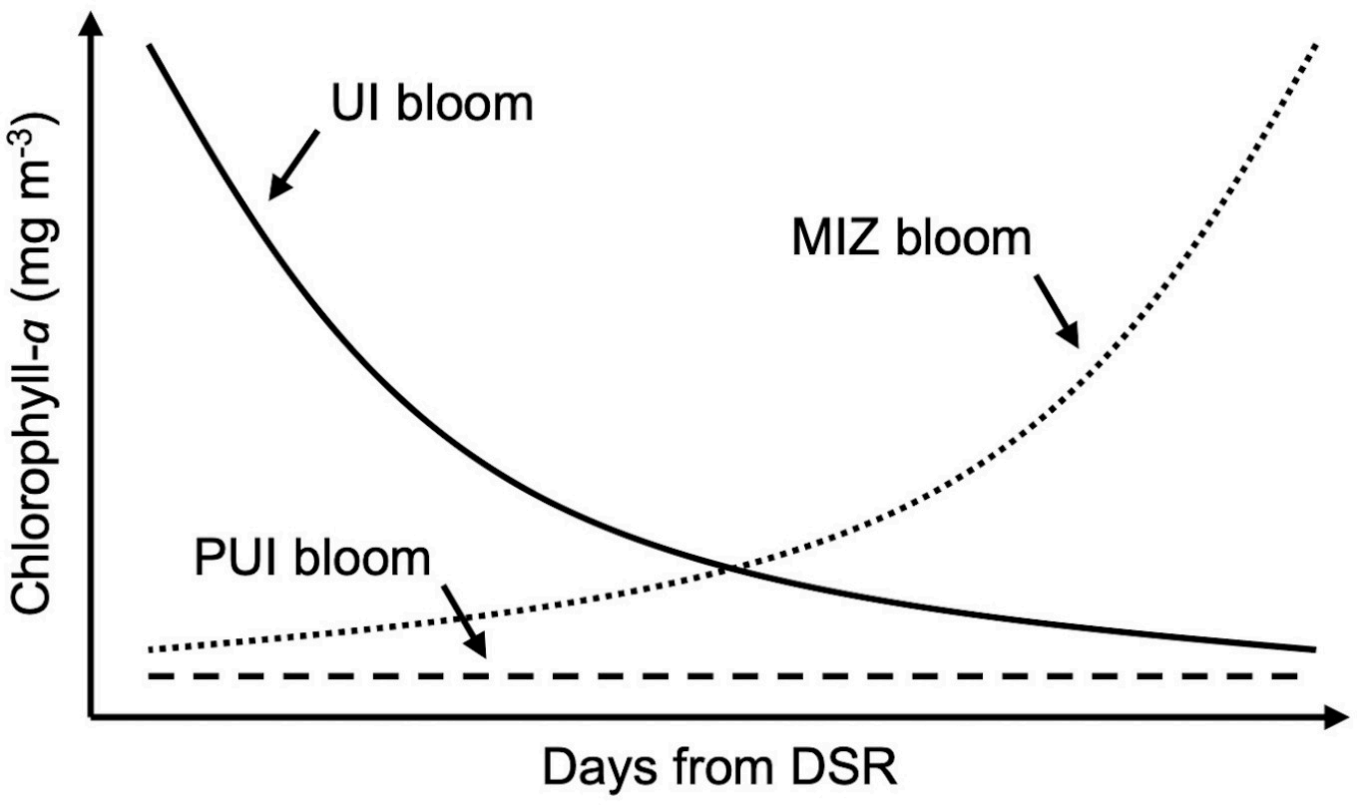
Conceptual diagram for three phytoplankton bloom types. Solid line, dashed line, and dotted line represent under-ice (UI) bloom, probable under-ice (PUI) bloom, and marginal ice zone (MIZ) bloom following the date of sea ice retreat (DSR), respectively. This conceptual diagram was modified from Lowry et al. [13].

Based on satellite-retrieved time-series of chlorophyll-*a* (chl*a*) concentration, which is a proxy of phytoplankton biomass at the sea surface, Lowry et al. [13] explored spatial patterns in spring phytoplankton bloom types in the Chukchi Sea. They classified spring bloom types into three groups, namely, UI blooms, probable under-ice (PUI) blooms, and MIZ blooms (Fig 1). UI blooms were defined as high chl*a* concentrations at the same time of the sea-ice retreat, indicating that there had occurred phytoplankton growth under the ice, whereas PUI blooms were defined as low chl*a* concentrations for the first several weeks of the ice-free period because UI blooms assumed to be matured and depleted nutrients before the ice retreat. MIZ blooms were characterized as low chl*a* concentrations at the time of sea-ice retreat and those chl*a* concentrations increased with time to have blooms days to weeks after the ice retreat. Using a simple bloom classification approach based on the timing when the satellite-derived chl*a* reached a bloom threshold of 2.5 mg m^−3^ following the retreating sea-ice edge, Lowry et al. [13] reported that earlier and later ice retreat will favor development of MIZ and UI blooms, respectively. In consequence, they established the critical relationship between the spring phytoplankton bloom types and timing of sea-ice retreat in the Chukchi Sea for the time period 1998–2012.

**Fig 2:**
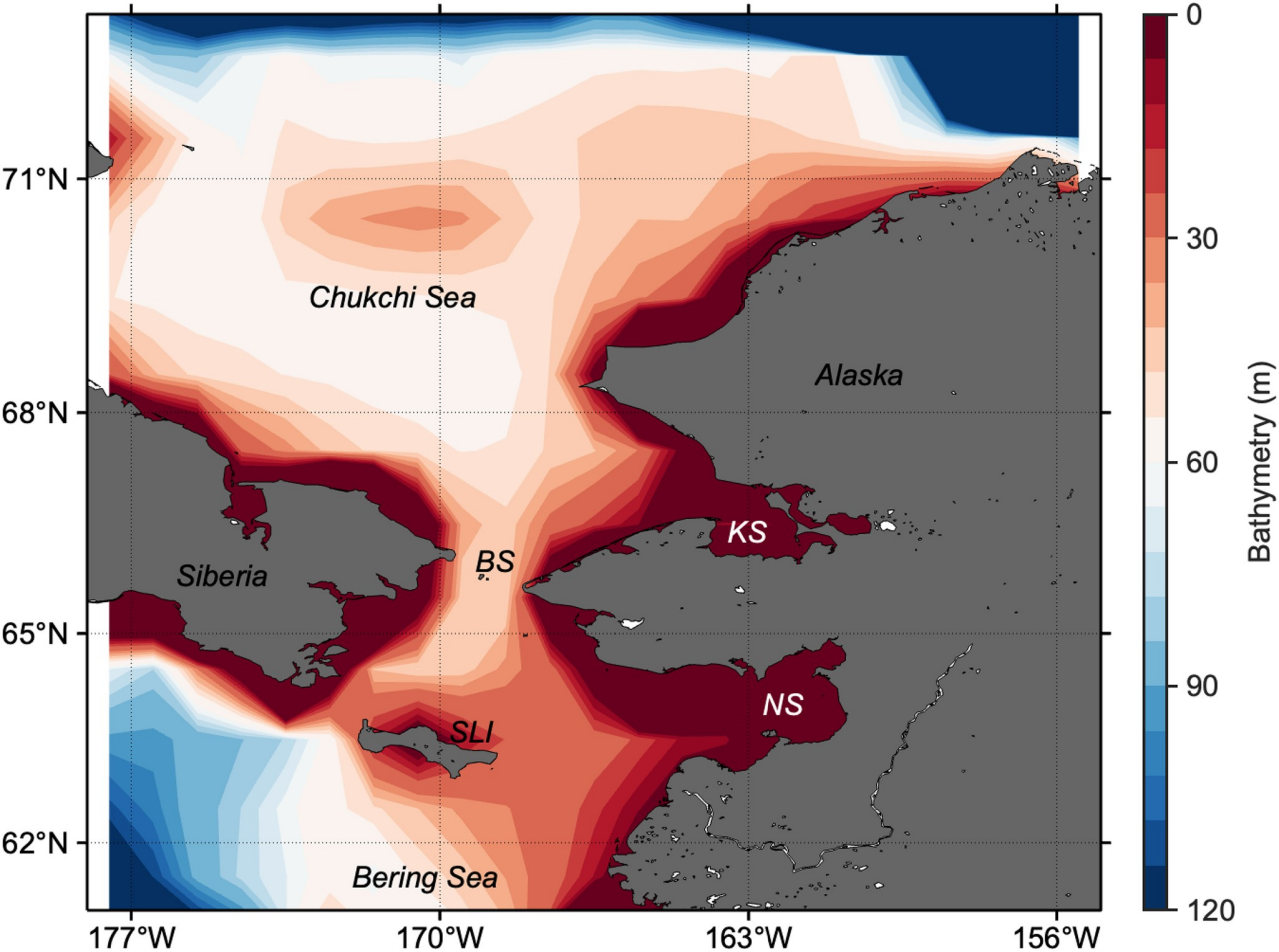
Bathymetry map of the Pacific Arctic. Abbreviations: SLI, St. Lawrence Island; BS, Bering Strait; NS, Norton Sound; KS, Kotzebue Sound.

The Pacific Arctic, specifically the region extending from the northern Bering Sea to the southern Chukchi Sea (Fig 2), is one of the most biologically productive regions in the world’s ocean and is currently experiencing drastic loss of seasonal sea ice [14, 15]. For example, the lowest winter sea-ice coverage on record (1980–2019) in the northern Bering Sea was observed in the winter of 2017–2018 [16]. The lack of ice in spring affected the temperatures and structure of the water column and, in turn, the timing of the spring bloom that can have cascading effects on the distribution of fish and the reproduction and survival of marine birds and mammals [17]. Recent work suggests that extreme events like those observed in 2018 may well become the norm as the Arctic and sub-Arctic warm [18]. In fact, anomalously warmer seawater temperatures were observed in the northern Bering Sea for 2017–2019 [19]. Additionally, the northern Bering Sea is of particular importance for economically valuable species such as salmon, crab, and groundfish [20]. Consequently, confirming the previously established critical relationship between the spring phytoplankton bloom types and timing of sea-ice retreat in the Chukchi Sea for 1998–2012 in the expanded time frame and study area contributes to a better prediction of how future changes in the Arctic would impact ice-associated phytoplankton blooms.

With the importance of accurate monitoring spring phytoplankton dynamics in a changing Arctic, this study examines the previously established critical relationship between the spring phytoplankton bloom types and timing of the sea-ice retreat for broader temporal and spatial coverages, with a particular focus on the Pacific Arctic for 2003–2019. To this end, time-series of satellite-retrieved phytoplankton biomass during the period of seasonal ice edge retreat were fit to an expected growth curve to identify the phase of the bloom using a parametric Gaussian function, as an effective approach to capture the development and decay of phytoplankton blooms [21–23]. The parametric Gaussian function has several advantages to retrieve information about phytoplankton bloom features, such as timing, amplitude, duration, and number of blooms within a given year [21–23]. Additionally, the parametric Gaussian function allows us to conduct sensitivity analyses examining the number of satellite-retrieved chl*a* required to distinguish accurately between spring phytoplankton bloom types [22]. Moreover, we can assess the statistical significance of the fitted parametric Gaussian function as a measure of errors and uncertainties. In the Arctic, a presence of extensive cloudiness obstructs satellite monitoring of the phytoplankton community [23]. Therefore, compared with the previous exploratory work drawing on a simple bloom type classification based on a chl*a* threshold [13], our approach offers the advantage of tracking the certainty/uncertainty of the fitted function as an uncertainty measure in capturing the development and decay of phytoplankton blooms.

The current study is a contribution to a special issue on the Distributed Biological Observatory (DBO), specifically to highlight the potential for satellite remote sensing in supporting point and transect-based measurements in a complex environment. The DBO is a change detection array for select ecosystem variables along eight sampling transects in the Pacific Arctic [24]. The overarching goal of the observatory is to provide for the detection and consistent monitoring of the biophysical responses to major reductions in seasonal sea ice and concomitant increases in seawater temperatures observed across the region. The standardized shipboard measurements made along the DBO transects focus on both lower and higher trophic levels. Classical methods relying on ship-based observations have been very successful to date in understanding local and snapshot of ocean [25] although these have difficulty to cover longer time-series and/or broader areas that requires a lot of costs, such as time, research funds, and efforts. This is where satellite techniques, such as those drawn on in the present study, offer extensive, sustained, and cost-effective observations. As a single cell of phytoplankton only lives for a few days and thus population and composition of phytoplankton community fluctuate strongly in a matter of days to weeks [26], satellite-based approaches provide a valuable asset for monitoring spatiotemporal dynamics of phytoplankton communities with appropriate spatial and time scales [27].

## Data and methods

### Satellite data

Level-3 daily standard mapped images of remote-sensing reflectance (*R*_rs_(*λ*)) at 9-km resolution at four wavelengths (*λ* = 412, 443, 488, and 555 nm), obtained by the Moderate Resolution Imaging Spectroradiometer (MODIS) sensor onboard the Aqua satellite, were downloaded from NASA’s Ocean Color website (https://oceandata.sci.gsfc.nasa.gov), for the period of 2003–2019. In addition, the Advanced Microwave Scanning Radiometer (AMSR) Unified daily sea-ice concentrations gridded at a 12.5-km resolution for the period of 2003–2019 were downloaded from the National Snow and Ice Data Center website (https://nsidc.org/). AMSR Unified sea-ice concentrations were created by reprocessing the Advanced Microwave Scanning Radiometer-Earth Observing System (AMSR-E) sensor onboard the Aqua satellite during 2003–2011 and Advanced Microwave Scanning Radiometer 2 (AMSR-2) sensor onboard the Global Change Observation Mission 1st-Water (GCOM-W1) during 2013–2019. To align the spatial resolution of satellite data used in this study, daily sea-ice concentrations were resampled onto the 9-km grid using nearest-neighbor interpolation [2]. Note that in 2012 there was a gap in temporal coverage during the transition from the AMSR-E to the AMSR-2 sensors. While this period of missing data could be filled in with data from other sensors, we exclude the year of 2012 from our analysis to minimize biases and uncertainties due to sensor-specific characteristics such as spatial resolution and algorithm.

### Chlorophyll-*a* retrieval

Chlorophyll-*a* (chl*a*) concentration was computed from *R*_rs_(*λ*) using the Arctic-OC3L algorithm developed and validated in the Chukchi Sea [28]. The Arctic-OC3L is a linear equation of the form below:

$$chla=10^{\left(a+bR\right)}$$ (1)

where the coefficients *a* and *b* are empirically derived values of 0.3364 and −3.4388, respectively, and *R* is the base-10 logarithm of the maximum band ratio of *R*_rs_(*λ*) among the three potential blue wavelengths (i.e., 412, 443, and 488 nm) and the green wavelength (i.e., 555 nm).

### Bloom-type classification

**Fig 3:**
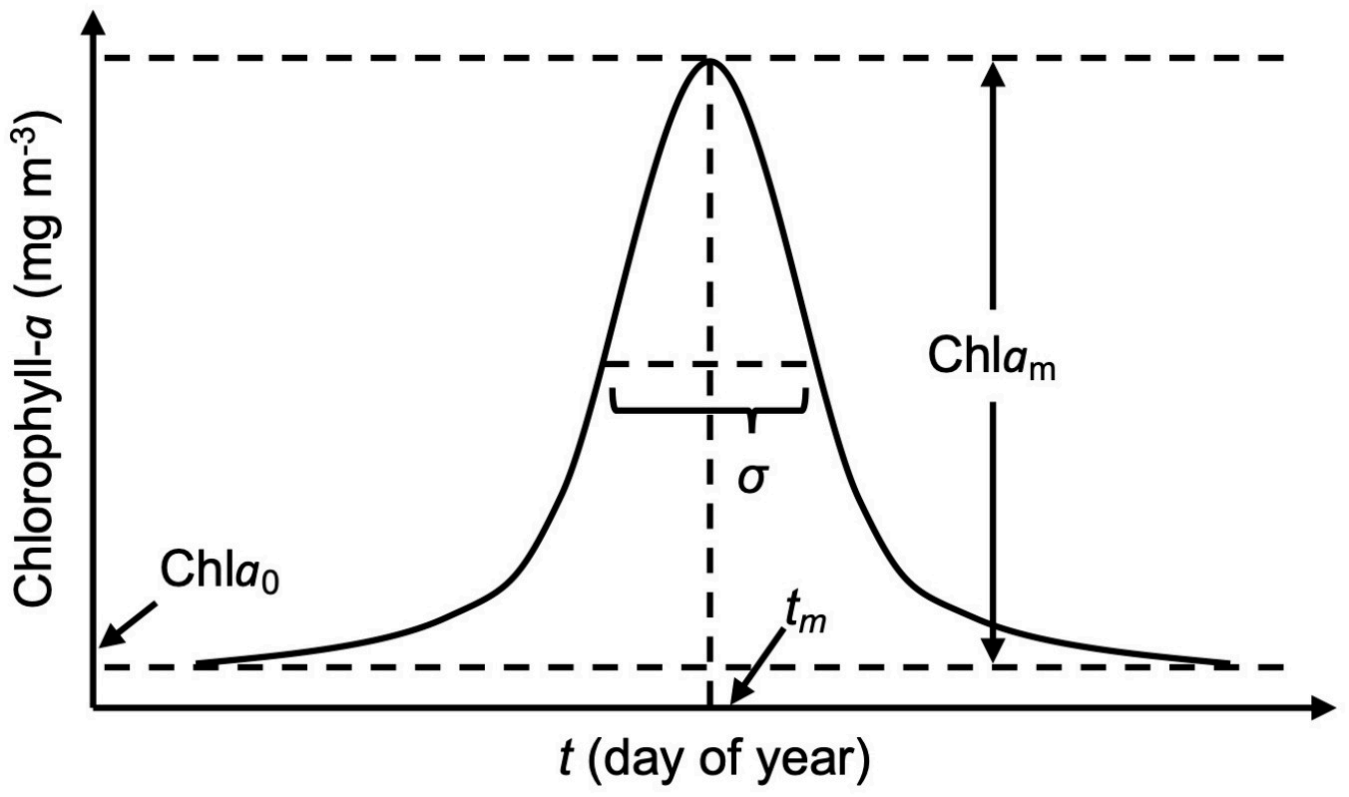
Description of the parametric Gaussian model. Abbreviations: chl*a*_0_, background chl*a*; chl*a*_m_, amplitude of chl*a* peak; *t*_m_, date of chl*a* peak; *σ*, days for which chl*a* exceeded half of chl*a* peak.

Time-series of daily chl*a* over a 20-day of MIZ retreat period following the date of sea-ice retreat (DSR) were fit to an expected growth curve to identify the phase of the bloom, i.e., UI, PUI, or MIZ bloom. It is noteworthy that the 20-day of MIZ retreat period was widely used in previous studies [2, 29, 30]. Here, DSR was defined as the first date in any year when sea-ice concentration fell below 50% [31], which is the best approximated ice edge visible in MODIS/Aqua quasi-true color images [3]. Additionally, sea-ice breakup was defined to occur some time during the period March 15–September 15 [14]. All pixels with a sea-ice concentration below 50% on March 15 were excluded from the subsequent analysis. To ensure that temporal variation in chl*a* was captured at a given location, phytoplankton blooms in spring were classified only as such if the pixel contained a minimum of one chl*a* value per week and at least one chl*a* concentrations within two days after onset of ice retreat [13]. The chl*a* time-series during the MIZ period was modeled as a Gaussian function using MATLAB *lsqnonlin* (R2020b, Optimization Toolbox) on a pixel-per-pixel basis as follows:

$$chla\left(t\right)={chla}_{0}+{chla}_{m}e^{\left(\frac{{\left(t-t_{m}\right)}^{2}}{2\sigma^{2}}\right)}$$ (2)

where *t* represents the date, chl*a*_0_ is the background chl*a*, chl*a*_*m*_ is the amplitude of the chl*a* peak observed on the date *t*_*m*_, and *σ* corresponds to the number of days chl*a*(*t*) exceeded half of chl*a*_*m*_ (Fig 3). Initial values, and lower and upper bounds of each variable in Eq (2) are shown in Table 1. These values were compared to in situ measured values of UI blooms in the Arctic [5, 6]. Note that MATLAB *lsqnonlin* with lower and upper bounds requires at least as many input data as the number of variables in these bounds such that the minimum number of chl*a* concentrations during the MIZ retreat period is five data points.

**Table 1:** Settings for the parametric Gaussian function.

Model parameter	Initial value	Lower bound	Upper bound
Background chl*a* (chl*a*_0_)	0.5 × chl*a*_MIZ_	0.0	1.0 × chl*a*_MIZ_
Amplitude of chl*a* peak (chl*a*_*m*_)	2.0 × chl*a*_MIZ_	2.5	30.0
Date of chl*a* peak (*t*_*m*_)	0.0	−30.0	30.0
Days exceeded the half of chl*a* peak (*σ*)	20.0	5.0	40.0

Then, the chl*a* time-series was assigned into any of three bloom types (Fig 1) based on following processes. The statistical significance of the fitted parametric Gaussian function was tested to the significance level of 5%. When the model was statistically significant (*p* < 0.05), the chl*a* time-series was classified into either an UI bloom or MIZ bloom. The distinction between UI and MIZ blooms is based on the model parameter *t*_*m*_: a chl*a* time series was classified as an UI blooms if *t*_*m*_ was observed before DSR (*t*_*m*_ < DSR); in contrast if *t*_*m*_ was equal to or after DSR (*t*_*m*_ ≥ DSR) the bloom was classified as a MIZ bloom. In the case that the models were statistically insignificant (*p* ≥ 0.05), chl*a* time-series is expressed as a constant at chl*a*_0_ as follows:

$$chla\left(t\right)={chla}_{0}$$ (3)

Modeled chl*a* time-series as a single term of chl*a*_0_ were classified as PUI blooms. This is based on the assumption that the pixels had already experienced a bloom before DSR, and thus the chl*a* retrievals during the MIZ retreat period were remnants of the UI bloom [13].

The classification was based on the assumption that pre-bloom surface nutrient concentrations are high throughout the Pacific Arctic, such that a phytoplankton bloom would be expected in waters with sufficient light availability. This assumption is reasonable because the vertical turbulent mixing of nutrient-rich Anadyr Water and Bering Shelf Water provide sufficient nutrients throughout the water column for phytoplankton growth in spring [32] and such nutrients would be depleted in cases phytoplankton blooms occur prior to ice retreat [33], suppressing the development of MIZ blooms and thus identified as PUI blooms [13].

### Sensitivity analysis

In the Arctic Ocean, the extensive cloud cover limits ocean-color data availability, and in turn may hamper parametric modeling of time-series variations in satellite-derived chl*a*. To ensure the robustness of our approach, we conducted a sensitivity analysis using the minimum required number (i.e., five) of chl*a* concentrations during the MIZ retreat period. First, a sample chl*a* time-series during the MIZ retreat period was generated by averaging daily chl*a* time-series during the MIZ retreat period for all pixels classified as UI blooms over the entire study period from 2003 through 2019. Then, five chl*a* concentrations were randomly selected from the sample chl*a* time-series as input data, and were modeled using the Eq (2). We repeated this treatment for 1000 trials, and then compared the resulting bloom type. Other sample chl*a* time-series during the MIZ retreat period for PUI and MIZ blooms were also generated to examine the consistency of the resulting bloom type.

Initial value, and lower and upper bounds of model parameters apply to Eqs (2) and (3). These values were determined on a pixel-by-pixel basis. Chl*a*_MIZ_ denotes averaged chl*a* during the MIZ retreat period for 2003–2019 on a pixel-by-pixel basis (see Fig 5B).

## Results

### Climatology of satellite-derived variables

**Fig 4:**
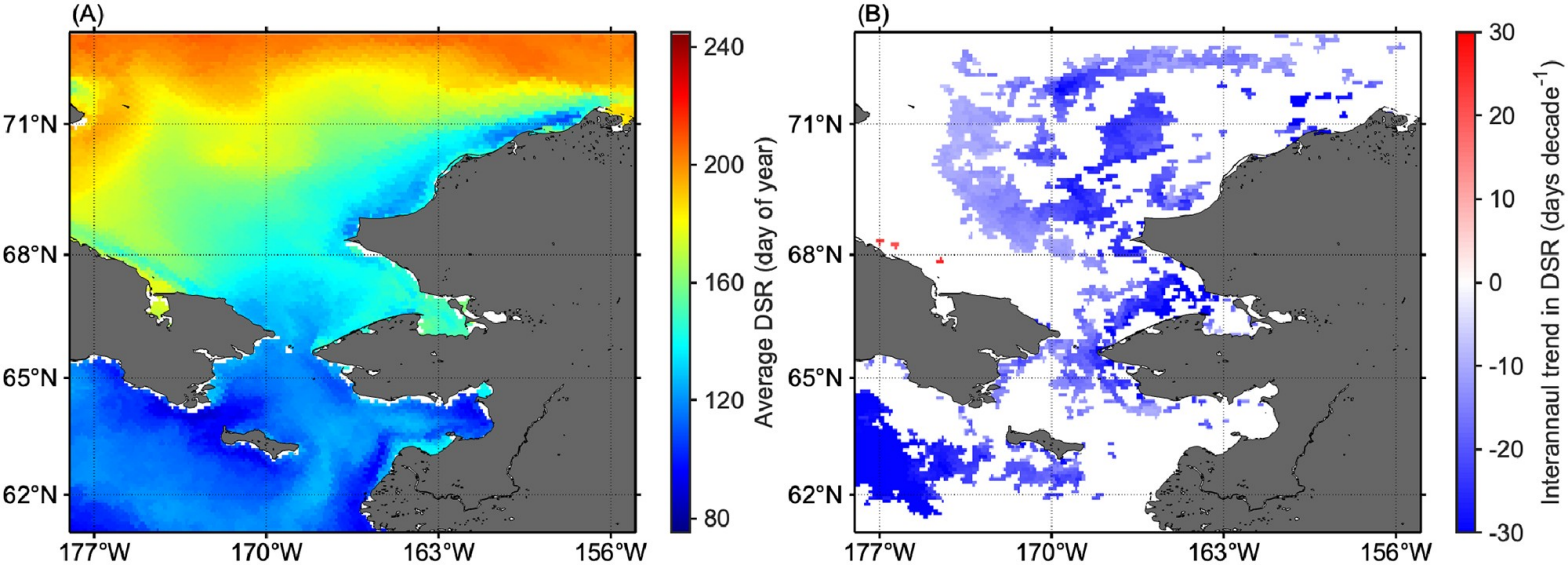
Spatial patterns in sea-ice climatology. (A) Average date of sea-ice retreat (DSR) and (B) interannual trend (Mann-Kendall test, *p* < 0.05) in DSR for 2003–2019. White represents areas with insignificant (Mann-Kendall test, *p* ≥ 0.05) temporal trend during the period.

Fig 4A shows the climatological average of DSR for 2003–2019. A clear latitudinal gradient is apparent with sea-ice retreat initiated in the south and advancing to the north between March and July. Although the DSR varied greatly throughout the Pacific Arctic during the study period, these south-north and east-west gradients in DSR were observed consistently across all years from 2003–2019. Furthermore, we found a statistically significant interannual trend in DSR (*p* < 0.05) for 43% of the grid cells in the study area (Fig 4B). The majority of these locations (99.7%) exhibited a significant negative trend, suggesting that the timing of sea-ice retreat shifts toward earlier dates over the time period 2003–2019.

**Fig 5:**
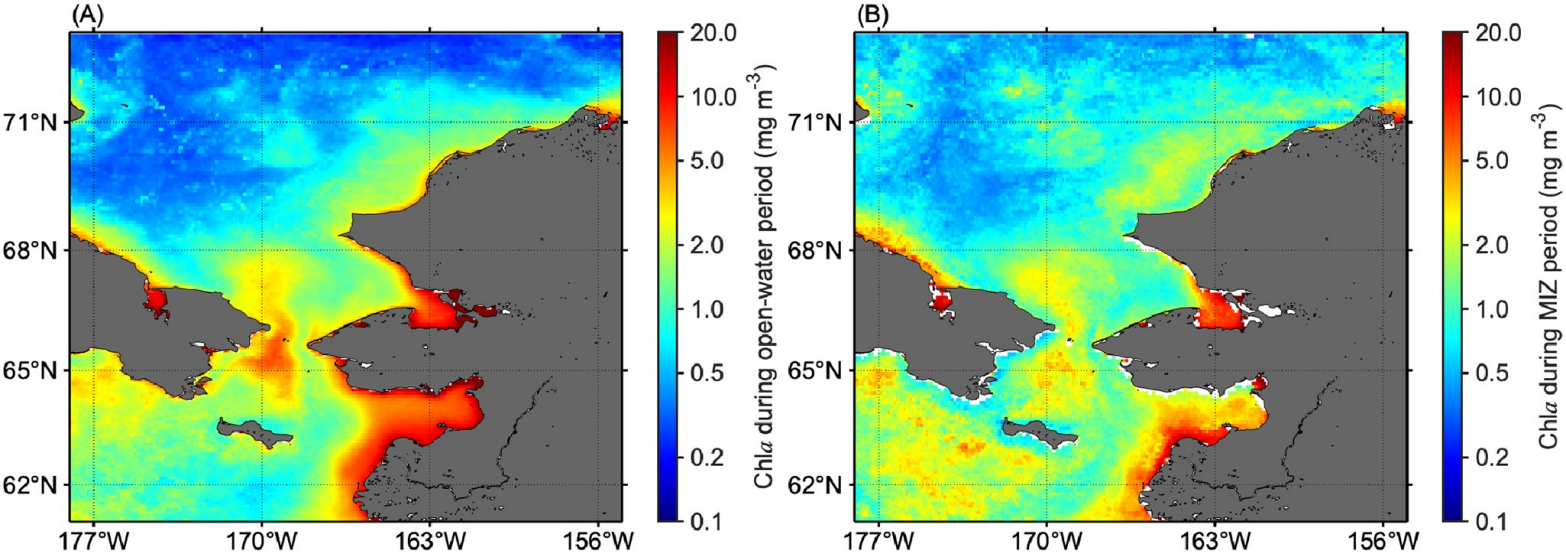
Spatial variations in average chlorophyll-*a* (chl*a*) concentrations for 2003–2019. (A) Entire open-water period (sea-ice concentration below 50%) and (B) marginal ice zone (MIZ) retreat period.

The satellite-derived chl*a* time-series throughout the year was averaged for 2003–2019 (Fig 5). High chl*a* values were found on the western side of Bering Strait which is highly influenced by nutrient-rich Anadyr Water; the eastern side of Bering Strait and south of St. Lawrence Island, where nutrient-poor Alaskan Coastal Waters dominate, exhibited lower chl*a* concentrations (Fig 5A). These spatial patterns were also found in average chl*a* fields during the MIZ retreat period (Fig 5B). However, higher chl*a* concentrations were observed during the MIZ retreat period across larger stretches of the Pacific Arctic than those found in the annually averaged chl*a* fields. Note that localized extreme concentrations of chl*a* associated with river deltas, estuaries and coastal lagoons, particularly in Norton Sound and Kotzebue Sound, are not necessarily indicative of biological production but may be artifacts due to high input of terrestrial organic matter and sediments.

### Chl*a* time-series for each bloom type

**Fig 6:**
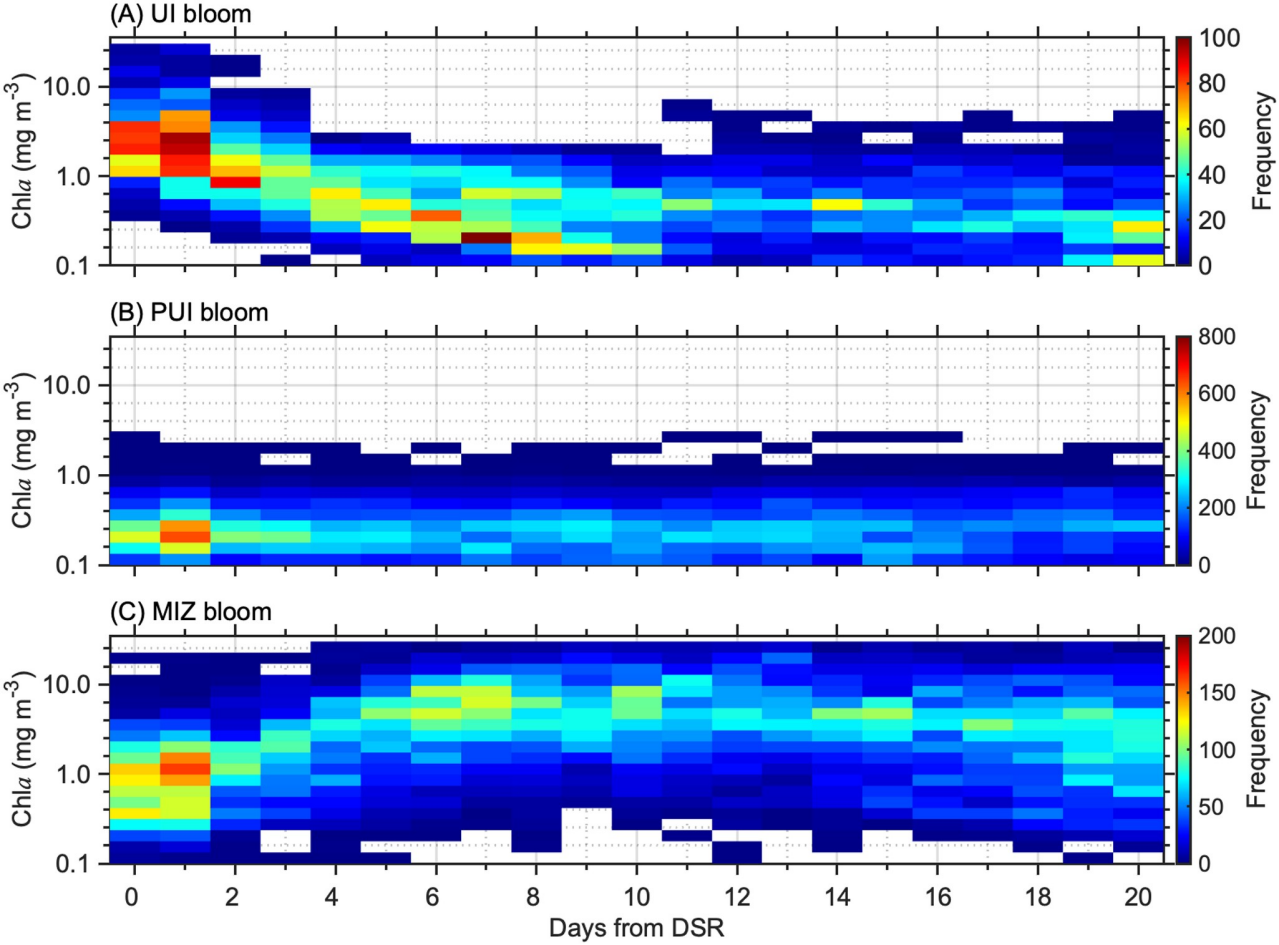
Frequency plots of collated chl*a* time-series during the MIZ retreat period. (A) UI blooms, (B) PUI blooms, and (C) MIZ blooms.

Chl*a* time-series during the MIZ retreat period were classified into one of the three specific types of blooms defined above by fitting the parametric model (Eqs 2 and 3). From this data, climatological chl*a* concentrations during the MIZ retreat period were derived for each bloom type by collating all pixels associated with one particular bloom for each progressive day after the DSR for 2003–2019. The collated chl*a* time series vary substantially between the three bloom types. UI blooms exhibited the highest chl*a* values within the few days after the DSR, subsequently decreasing over time (Fig 6A) probably because the UI blooms had already matured before the DSR. Chl*a* time series associated with PUI blooms maintained low values throughout the MIZ retreat period without significant fluctuations (Fig 6B). MIZ blooms exhibited chl*a* concentrations that increased from low to high values over the course of 4–5 days, maintaining high levels for a week or more (Fig 6C).

### Robustness of chl*a* time series parametric model

**Fig 7:**
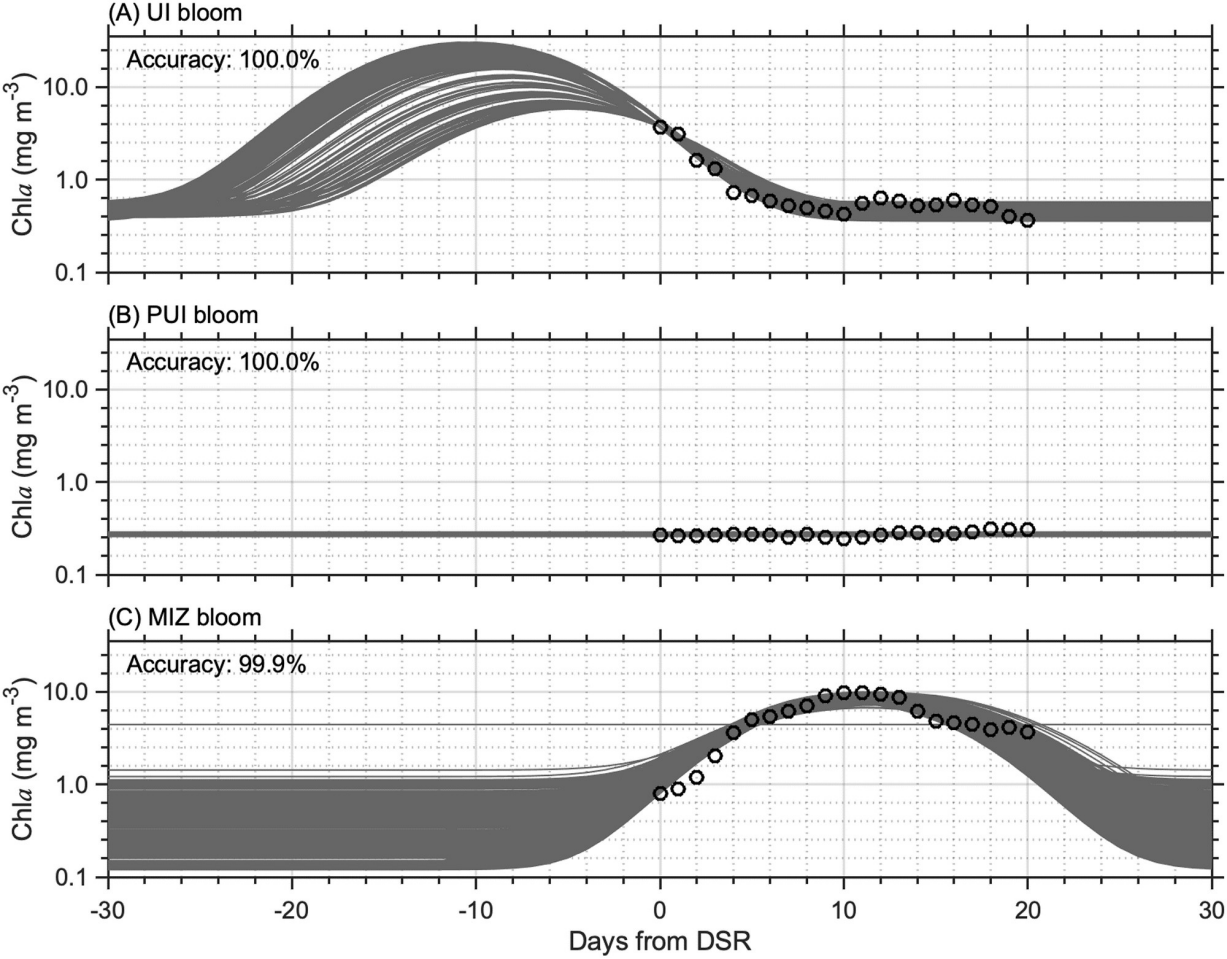
Results of the sensitivity analysis for the bloom type classification. (A) UI, and (B) PUI, and MIZ blooms. Modeled chl*a* time-series from 30 days prior to DSR until 30 days after DSR using five randomly-selected chl*a* concentrations during the MIZ retreat period are shown as gray lines. Black circles represent sample chl*a* time-series during the MIZ retreat period. The accuracy indicates the rate of correct identification for each bloom type.

The results of our sensitivity analysis are shown in Fig 7. Fitting the Gaussian function to five randomly-selected chl*a* concentrations out of 21 chl*a* concentrations (black circles in Fig 7) over the MIZ retreat period resulted in correct identification of UI (Fig 7A), PUI (Fig 7B), and MIZ blooms (Fig 7C) with accuracies of higher than 95% for each bloom type, indicating that our parametric modeling approach distinguished the timing of bloom correctly. However, other model parameters (i.e., bloom amplitudes and durations, and background chl*a*) for UI and MIZ blooms varied substantially, suggesting this parametric modeling approach induces substantial uncertainties in estimating these bloom characteristics. In contrast, temporal variations in chl*a* for PUI blooms were accurately fitted by the parametric approach, because this bloom type can be expressed by a single model parameter (i.e., background chl*a*).

### Spatial patterns in spring bloom types

**Fig 8:**
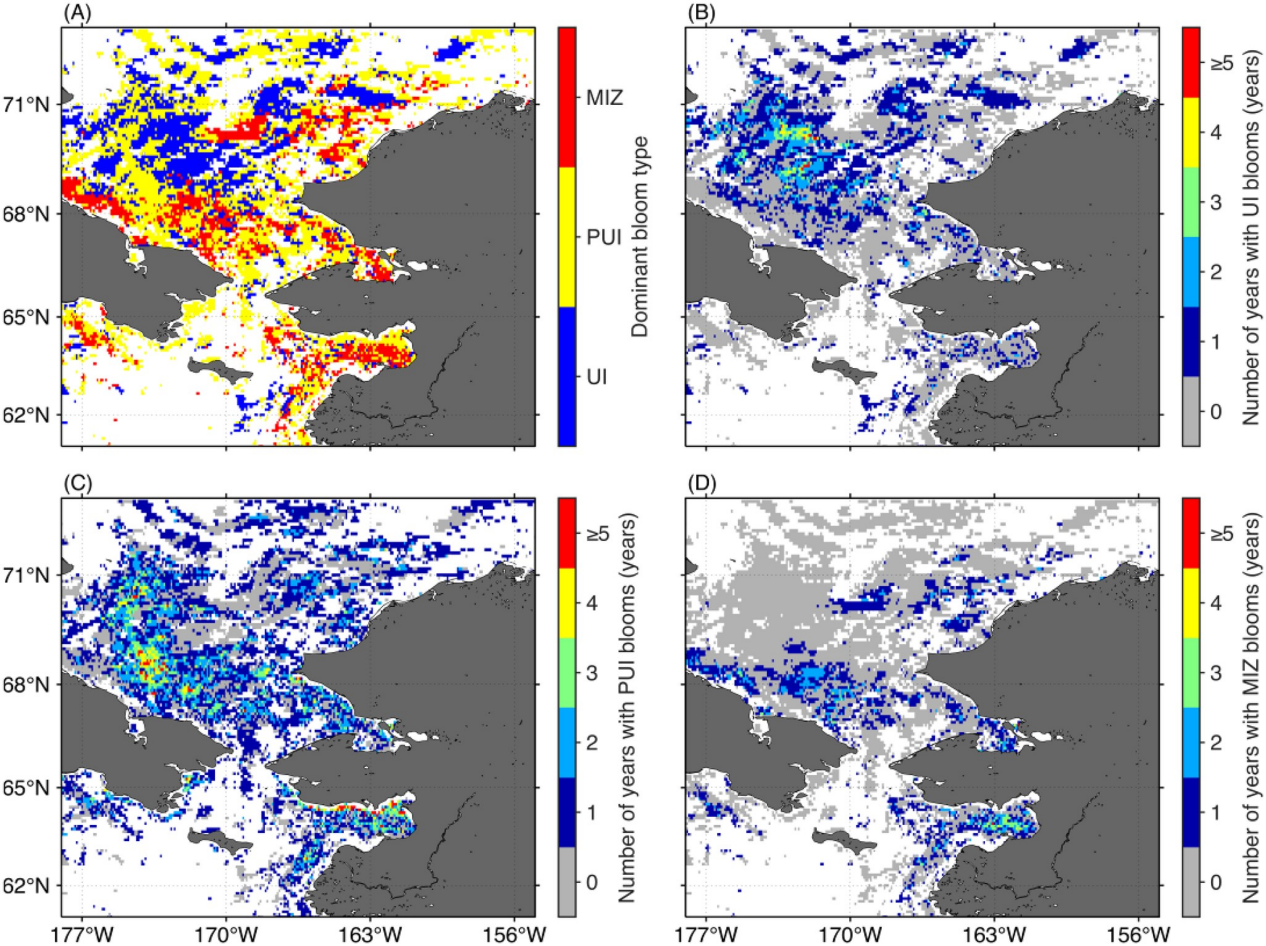
Spatial distribution of spring phytoplankton bloom types. (A) Dominant bloom type, and number of years from 2003 through 2019 with observations of (B) UI, (C) PUI, and (D) MIZ blooms. White represents areas where none of the years 2003–2019 satisfied our criteria.

Proportions of each bloom type in individual years are summarized in Table 2. The most favorable bloom type in the study area was the PUI bloom (58.7 ± 7.0%), followed by UI (24.5 ± 10.5%) and MIZ blooms (16.9 ± 8.5%). It is important to note that there was no significant relationship between the proportions of pixels satisfying the parametric model criteria and the bloom types at those pixels (UI bloom, *p* = 0.14; PUI bloom, *p* = 0.05; MIZ bloom, *p* = 0.75). Fig 8A shows the spatial distribution of the dominant bloom type for the time period 2003–2019. When we generated maps representing the total number of years with a specific bloom type (Fig 8B–8C), the resulting maps show that each bloom type occurred across the entire Pacific Arctic sector. Particularly, the number of years with UI blooms was disproportionately higher in the Chukchi Sea than in other areas (Fig 8B). Indeed, the UI bloom was the dominant bloom type in a substantial portion of the Chukchi Sea (Fig 8A). PUI blooms registered as the dominant bloom types in a wide area of the Pacific Arctic (Fig 8A), whereas the MIZ bloom was the relatively minor bloom type across the Pacific Arctic.

**Table 2:** Summary statistics for proportions of each bloom type in the Pacific Arctic.

Year	DSR	Observable area	UI bloom	PUI bloom	MIZ bloom
	(day of year)	(% ocean surface)	(% area)	(% area)	(% area)
2003	146.6 ± 33.5	12.6	49.4	42.9	7.7
2004	152.0 ± 38.6	4.5	35.4	58.7	5.9
2005	153.1 ± 36.6	12.3	37.5	54.0	8.5
2006	165.0 ± 40.3	8.4	23.8	67.4	8.8
2007	146.6 ± 31.8	5.5	25.8	64.2	10.1
2008	160.3 ± 31.7	2.8	21.3	63.5	15.2
2009	156.3 ± 26.6	7.7	23.1	48.9	28.0
2010	157.6 ± 33.7	2.5	11.4	62.0	26.6
2011	144.6 ± 35.3	7.4	16.6	69.9	13.5
2013	159.2 ± 30.7	1.3	12.0	60.3	27.7
2014	151.4 ± 37.7	5.1	32.4	57.4	10.3
2015	144.3 ± 33.7	13.8	28.4	51.4	20.2
2016	149.4 ± 38.8	14.6	23.3	55.6	21.1
2017	136.1 ± 35.8	9.6	21.0	59.8	19.2
2018	130.5 ± 44.9	8.2	8.5	58.3	33.2
2019	125.2 ± 40.7	6.4	21.4	64.4	14.1
2003–2019	148.6 ± 35.6	7.7 ± 4.1	24.5 ± 10.5	58.7 ± 7.0	16.9 ± 8.5

Date of sea-ice retreat (DSR), observable area, and proportions of under-ice (UI) bloom, probable under-ice (PUI) bloom, and marginal ice zone (MIZ) bloom; average ± standard deviation.

### Spring bloom types and timing of sea-ice retreat

**Fig 9:**
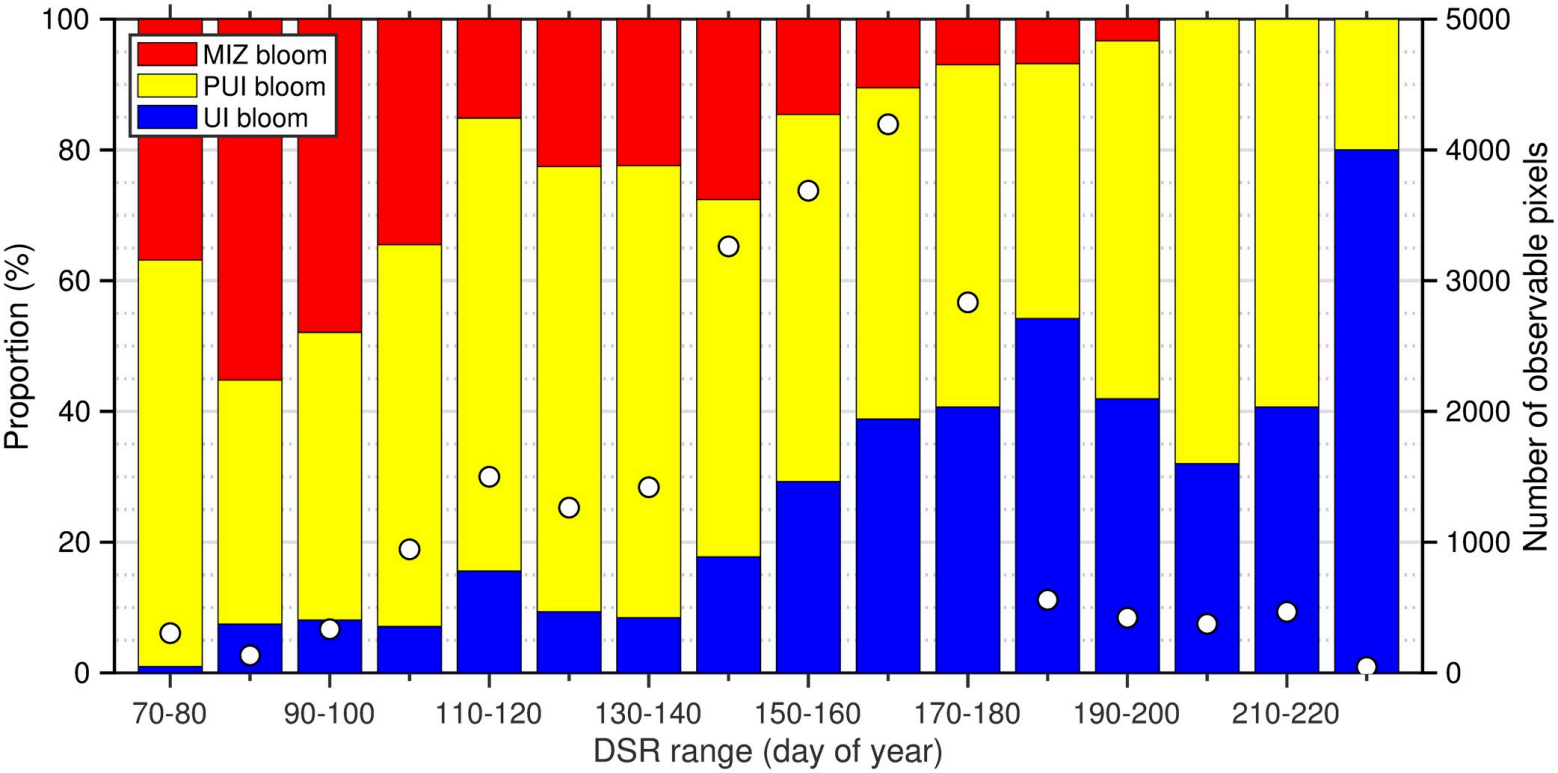
Proportion of each bloom type and number of observable pixels within ten-day DSR range steps. Blue, yellow and red bars represent proportions of UI, PUI, and MIZ blooms, respectively. White dots indicate number of observable pixels.

**Fig 10:**
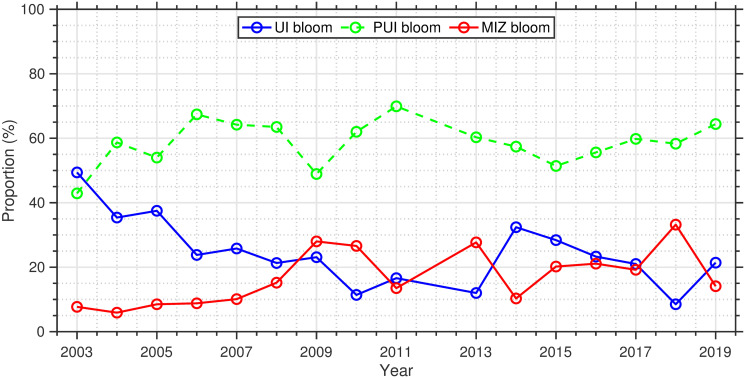
Interannual variations in proportions of each bloom type for 2003–2019. Solid and dashed lines represent statistically significant (*p* < 0.05) and insignificant trends, respectively.

As we found specific spatial patterns both in the bloom types and DSR, the potential influence of DSR on the bloom type were examined. We found clear linkages between bloom type and DSR (Fig 9): later and earlier DSR support UI and MIZ blooms, respectively, whereas occurrences of PUI blooms did not appear to correlate with DSR. This finding suggests that the trend towards earlier DSR can drive interannual shifts in the bloom type. In fact, we found significant (*p* < 0.05) decreasing and increasing trends in the proportions of UI blooms and MIZ blooms for 2003–2019 (Fig 10). In addition, DSR exhibited significant (*p* < 0.05) positive and negative correlations with the proportions of UI and MIZ blooms, respectively. These findings demonstrate the influence of interannual variations in DSR on bloom typology in the Pacific Arctic. Note that the mean proportion of pixels satisfying the parametric model criteria for 2003–2019 was 7.7 ± 4.1% of ocean surface in the Pacific Arctic (Table 2), suggesting the relative proportions of bloom types and their interannual trends might have substantial uncertainties.

## Discussion

### Impacts of DSR on spring phytoplankton bloom dynamics

The present study examined the spatiotemporal variations in spring phytoplankton bloom types and their relationship with the progression of sea-ice retreat in the Pacific Arctic for the time period 2003–2019. We confirmed that the critical relationship between the spring bloom types and timing of the sea-ice retreat previously established by Lowry et al. [13] for the Chukchi Sea for 1998–2012 extend across the entire Pacific Arctic, i.e., including in particular the Bering Sea, for the time period 2003–2019 (Fig 9). Our results, derived through use of a parametric model (as opposed to a fixed chla threshold) with broader temporal and spatial coverage strengthen and expand their findings of recent shifts in phytoplankton bloom types in the Chukchi Sea [13]. Overall, this study provided additional evidence about impacts of a shift in sea-ice retreat timing on spring phytoplankton blooms.

Over the western Chukchi shelf, which experiences a longer duration of ice cover (Fig 5A), high shortwave fluxes during the melt season and the presence of melt ponds which increases the illumination of the water column beneath the sea ice [12, 34] likely fostered a favorable under-ice light regime. Indeed, UI blooms were frequently observed across this area (Fig 8B). Conversely, MIZ blooms were the predominant bloom type in some portions of the other areas (Fig 8D). A reasonable explanation for the predominance of MIZ blooms is the trend towards earlier sea-ice retreat [13], because earlier sea-ice retreat not only promotes light penetration into the water column but also convective overturning driven by refreezing of surface waters in sea-ice leads [35]. As convective mixing is active during springtime and then gradually subsides towards summer [36], the earlier sea-ice retreat could promote convective mixing to deeper depths that reduces the light phytoplankton receive [35]. Likewise, if the sea-ice retreats before the last of the winter storms, strong wind mixing delays the start of the spring blooms until insolation warms the upper water column sufficiently to prevent mixing by stratification [37, 38]. These two processes would result in the delayed development of the spring phytoplankton blooms (i.e., MIZ blooms). Overall, this study demonstrated the critical relationship between the spring boom types and timing of the sea-ice retreat as reported by Lowry et al. [13].

Regarding the influence of timing of sea-ice retreat on spring phytoplankton dynamics, several studies have partially examined this relationship using satellite-based approaches. For example, Brown and Arrigo [30] reported that ice-edge blooms during late sea-ice retreat years were less productive while open water blooms were observed in early sea-ice retreat years. They also reported that open water blooms were approximately 26% more productive than ice-edge blooms. Additionally, Fujiwara et al. [39] reported that phytoplankton communities during the MIZ period were associated with the dominance of larger and smaller phytoplankton in earlier and later sea-ice retreat years, respectively. While the dominance of spring phytoplankton blooms in the Arctic had been attributed previously to increased light availability and water column stratification following sea-ice retreat, the discovery in 2011 of a massive phytoplankton bloom underneath first-year sea ice in the Chukchi Sea shows current understanding of Arctic marine ecosystems may need to be revised in parts [3]. In this context, previous findings of the impacts of the timing of sea-ice retreat on phytoplankton dynamics during the MIZ period may have to take the presence of UI blooms into account; i.e., the ice-edge blooms likely to be remnants of UI blooms that consume surface nutrient concentrations prior to sea-ice retreat and thereby large-celled phytoplankton cannot thrive in nutrient-limited waters in the MIZ period.

### Long term trend in spring phytoplankton bloom types

Long-term analysis of the sea-ice concentration revealed that a substantial portion of the Pacific Arctic has been experiencing a shift in the timing of sea-ice retreat toward earlier dates (Fig 5B), as has been reported in a previous study [8]. Considering that the timing of sea-ice retreat was shown here to potentially alter the type of the spring phytoplankton blooms in the study area (Fig 9), interannual variations in the timing of sea-ice retreat would have influenced spring phytoplankton bloom typology [13]. In fact, associated with a clear linkage between the timing of sea-ice retreat and spring phytoplankton bloom types (Fig 9), we found significant decreasing and increasing trends (*p* < 0.05) in the proportions of UI and MIZ blooms accompanied by the changing timing of sea-ice retreat for the period 2003–2019 (Fig 10; Table 2). As the recent trend in the timing of sea-ice retreat is expected to continue in the future, the proportions of UI and MIZ blooms are likely to exhibit continued decreasing and increasing trends in the Pacific Arctic. It is noteworthy that the proportion of MIZ blooms in the Pacific Arctic has increased from 2003 through 2019, still approximately 50% of the study region would support UI and PUI blooms. These facts indicate that primary production underneath sea ice still plays a vital role in marine ecosystems in the recent Pacific Arctic. Therefore, the contribution of primary production underneath the ice must be included in estimates of total primary production [4, 40].

Lowry et al. [13] have reported a statistically increasing trend in the proportion of MIZ blooms from 1998 to 2012 in the Chukchi Sea, whereas there was no significant trend in UI blooms during this time period in the area. These results are partly consistent with the present study, which demonstrated significant increasing and decreasing trends in the proportion of MIZ and UI blooms, respectively, in the Pacific Arctic for 2003–2019. In 2017, thermal regimes in the Pacific Arctic showed signs of a sudden and dramatic shift relative to historical means and even to other recent unusually warm years [41]. In fact, average DSR over the Pacific Arctic showed exceptionally small values in the extreme years of 2017–2019 (Table 2). During these extreme years, particularly in 2018, we found smaller and larger proportions of UI and MIZ blooms, respectively, presumably associated with the critical relationship between the spring boom types and timing of the sea-ice retreat [13]. When comparing the study periods between the present (2003–2019) and previous studies (1998–2012), our study covering the extreme thermal phase in 2017–2019 is likely to yield significant results in trend analysis because a relatively short time series is prone to endpoint bias. Since extreme events may well become the norm as the Arctic warms [18], our trend analysis would have reflected reasonable shifts in spring phytoplankton bloom typology in the changing Pacific Arctic. It is important to recall that the mean proportion of pixels satisfying the parametric model criteria for 2003–2019 was 7.7 ± 4.1% of the ocean surface in the Pacific Arctic (Table 2). The relative proportions of bloom types and their interannual trends might have uncertainties because spatial biases in the observable areas for each year disturbed an accurate comparison.

Phytoplankton blooms in the Arctic have typically been tied to the retreating ice edge as enhanced growth of phytoplankton populations occurs along the seasonally retreating ice edge [1]. However, this paradigm may not be appropriate for a rapidly changing Pacific Arctic and neither for other Arctic regions experiencing such change. Horvat et al. [42] demonstrated that the recent transition from thick multiyear ice to thin ponded first-year ice permits more light penetration beneath the ice in the Arctic Ocean. In addition, Ardyna et al. [5] highlighted increasing light availability beneath sea ice associated with diminished regions of compact pack ice, defined as the area with ice concentration greater than 80%; this more favorable light regime allows for net phytoplankton growth and biomass increases on a pan-Arctic scale. These studies clearly suggest that phytoplankton phenology in the pan-Arctic is now experiencing a shift from MIZ blooms to UI blooms. Interestingly, phytoplankton blooms appear to exhibit opposing trends in the Pacific Arctic (Fig 10), including the Chukchi Sea [13], and pan-Arctic region [5, 42]. One potential explanation is that the timing of ice retreat in the Pacific Arctic has shifted to dates that are too early in the year to support phytoplankton growth even under highly ponded thin ice. Accompanying a sustained shift of sea-ice retreat timing toward earlier dates across the pan-Arctic, the current shift from MIZ blooms to UI blooms at high Arctic latitudes could eventually result in an opposite shift from UI blooms to MIZ blooms, as observed in the Pacific Arctic for 2003–2019.

### Relevance with the exploratory work

The foundation of this study has been built by Lowry et al. [13], who explored variations in spring phytoplankton bloom types in the Chukchi Sea for 1998–2012. One methodological difference from Lowry et al. [13] is our use of a parametric Gaussian function for capturing time-series variations in satellite-derived chl*a* at a given pixel [21–23]. The parametric Gaussian function has often been used for monitoring phytoplankton community dynamics as an effective approach to capture the development and decay of phytoplankton blooms. For example, Ardyna et al. [21] reported the shifts from a single spring bloom to double phytoplankton blooms (both spring and fall) in the pan-Arctic accompanied by an increasing trend in the number of stormy days over the open-water area. In addition, Marchese et al. [23] investigated interannual changes in spring-bloom characteristics in the North Water polynya in northern Baffin Bay. Furthermore, Waga and Hirawake [22] explored interannual variations in occurrences of evident fall blooms in the Pacific Arctic. Our sensitivity analysis demonstrated accurate estimates of timing and presence/absence of chl*a* peak even with some missing values, whereas other bloom features such as amplitude and duration likely contain large uncertainties (Fig 7A and 7C). Overall, the parametric Gaussian function is a powerful tool for examining the timing and presence/absence of phytoplankton blooms based on satellite-retrieved chl*a* time series in the Pacific Arctic and other Arctic seas.

Based on the presence/absence and the timing of chl*a* peaks captured by the parametric Gaussian function, the chl*a* time-series was classified into any of three spring bloom types originally proposed by Lowry et al. [13]. In the Pacific Arctic, convective wintertime mixing due to brine rejection [43] as well as northward advection of nutrient-rich waters from the Bering Sea [44] sustain very high nutrient concentrations in early spring [32]. Such nutrients would have been depleted if phytoplankton blooms had occurred prior to ice retreat [33], suppressing the development of MIZ blooms and thus leading to a classification as PUI blooms. In fact, UI blooms showed the highest chl*a* concentrations just after onset of sea-ice retreat which subsequently decreased monotonically with time (Fig 6A). This pattern is likely the result of surface nutrient depletion by mature phytoplankton blooms beneath the ice prior to ice retreat. In addition, vertical nutrient supply would have been suppressed by increased water column stratification associated with melting snow and sea ice, resulting in insufficient nutrients for phytoplankton growth during the MIZ retreat period. On the other hand, chl*a* time-series for MIZ blooms exhibited an evident peak as a result of rapid increase in phytoplankton population after the onset of ice retreat (Fig 6C). Areas without an evident chl*a* peak during the ice retreat period were considered to have experienced nutrient consumption and depletion prior to ice retreat by mature UI blooms (Fig 6B). According to Perrette et al. [2], ~90% of spring phytoplankton blooms occur within 20 days after onset of ice retreat in the Arctic. In addition, spring chl*a* peaks were observed within 20 days after the sea-ice retreat in a substantial portion of the Pacific Arctic [39]. Thus, this study extended the MIZ retreat period to a total of 20 days subsequent to the DSR, in contrast with a MIZ retreat period defined by the 14 days subsequent to the DSR as defined by Lowry et al. [13]. This extended time period should have captured PUI blooms in a realistic fashion; however, PUI blooms might include areas that experienced open-water blooms without UI or MIZ blooms.

Moreover, the present study expanded both the study region and included more recent data compared to Lowry et al. [13], who addressed interannual variations in spring bloom types using a satellite-based approach in the Chukchi Sea for 1998–2012. The Pacific Arctic, including not only the Chukchi but also the Bering Sea, has been facing the drastic changes in sea-ice fields, particularly in the recent several years [45–47]. Furthermore, the Bering Sea supports one of the largest and most profitable commercial fisheries, such as salmon, crab, and groundfish [20]. With the expanded time frame and study area, we find that the relationship between spring boom types and timing of sea-ice retreat previously established by Lowry et al. [13] holds over a larger region. Such further evidence of the how changing sea-ice retreat timing can drive variations in phytoplankton bloom dynamics can help contribute to better prediction of marine ecosystem shifts in the Arctic in the future.

### Potential uncertainties of a satellite-based approach

Both our study and Lowry et al. [13] do not take into accout the potential impacts of lateral advection because time series of satellite-retrieved chl*a* concentrations at each pixel were assumed to reflect variations of phytoplankton biomass within the same water body. Average flow speed over the Bering and Chukchi shelves is ~5 cm s^−1^ [48, 49], corresponding to an advection distance of ~86 km over the MIZ retreat period. Considering that the spatial correlation lengths in the phytoplankton community are at least more than 100 km in the global ocean [50], the water advected into a pixel during the MIZ retreat period is likely of a similar chl*a* concentration as the water that is being replaced. Thus, the potential impacts of lateral advection would likely not invalidate the classification of spring phytoplankton blooms based on our approach [13].

Sea-ice related error impacts on satellite-retrieved chl*a* concentrations are other potential weaknesses of satellite-based approach as discussed in Lowry et al. [13]. The sea-ice impacts are grouped into two components [51]: the adjacency effect that occurs along the boundary between sea ice and open water, and the sub-pixel contamination caused by the presence of sea ice within an ocean pixel. Regarding the satellite-retrieved chl*a* concentrations, the adjacency effect can lead to an over- and under-estimation at low concentrations (<0.05 mg m^−3^) and at high concentrations (>0.5 mg m^−3^) within a distance of ~10–20 km from the ice edge, respectively, whereas the sub-pixel contamination results in overestimation at moderate to high concentrations (>0.05 mg m^−3^). Therefore, the adjacency effect could lead to an underestimation of UI blooms, whereas the sub-pixel contamination could lead to an overestimation of UI blooms. By comparing the resulting proportions of three bloom types using a more conservative ice retreat threshold, Lowry et al. [13] demonstrated that the proportion of UI blooms increased from 11.6% to 14.3% when the ice threshold was lowered to 10% from 50%. Likewise, we also found slight increases in the proportions of UI blooms from 24.5 ± 10.5% to 28.6 ± 10.6% by lowering the ice threshold to 10% from 50%. As a lower ice threshold would have resulted in fewer mixed-ice ocean pixels and a greater distance between observable pixels and the adjacent ice edge, these results clearly suggest that the UI blooms are not subject to overestimation due to the sub-pixel contamination resulting from the 50% ice threshold [13]. Although the influence of the adjacency effect has not been completely ruled out by these analyses, we found a clear variation in proportions of spring bloom types in response to the timing of sea retreat (Fig 9) that suggests that sea-ice impacts on satellite chl*a* retrievals are less significant in determining spring bloom types.

The magnitude of the zooplankton grazing on phytoplankton growth is primarily controlled by grazer biomass which was very low in spring, when zooplankton was low [52, 53]. Indeed, Campbell et al. [53] estimated that more than half of the daily water-column primary production is not grazed by zooplankton communities at bloom locations in the Pacific Arctic. Driven by shifts from UI blooms to MIZ blooms, the Pacific Arctic food web is likely to undergo a transition from pelagic-benthic to pelagic-oriented systems [54]. Yet, the grazers are unable to control prodigious phytoplankton population growth that was observed during spring blooms [55], suggesting that zooplankton grazing is unlikely to significantly impact the results of our analysis.

Our sensitivity analysis demonstrated a robust performance of the parametric approach in identifying spring phytoplankton bloom types in the Pacific Arctic (Fig 7). However, this study did not calibrate or validate the parametric approach with actual field data. In addition to the aforementioned methodological uncertainties, satellite retrievals of chl*a* concentration may be subject to errors due to the optical complexity of the Arctic waters [56]. Moreover, extensive cloud cover often obstructs continuous satellite monitoring of the phytoplankton community in the Arctic [23], hampering the parametric approach which requires time series of chl*a* concentrations. Therefore, calibrating/validating the performance of our approach with in situ time-series data, such as at a mooring location, would further help assess the broader validity of these satellite-based findings. It is important to reinforce that this study provided an important insight into satellite-based monitoring of phytoplankton communities in the Pacific Arctic, which can contribute to developing and managing plans and strategies for in situ observation and/or related field activities in the future.

## Conclusions

Although monitoring phytoplankton communities using satellite always contain uncertainties, the present study demonstrated robust performance of a parametric, Gaussian fitting function for the satellite time series of phytoplankton biomass in identifying spring phytoplankton bloom types (Fig 7). Through the satellite-based parametric approach, this study established that the previously derived critical relationship between the spring phytoplankton bloom types and timing of the sea-ice retreat [13] holds for the broader Pacific Arctic region. Additionally, associated with a shift of sea-ice retreat timing toward earlier dates, this study identified shifts towards less frequent under-ice and more frequent marginal ice zone blooms in the Pacific Arctic (Fig 10). As the overarching goal of the DBO is to detect and monitor the biophysical responses to major environmental variations in the Pacific Arctic, the present study contributes to the DBO framework by providing additional evidence of the changing sea-ice retreat timing that can drive variations in phytoplankton bloom dynamics. Since the timing of biomass settling out of phytoplankton blooms is a key factor in many marine organism lifecycles [57, 58], monitoring variations in phytoplankton blooms is one of the crucial factors required for an improved holistic understanding of marine ecosystem variability and transitions. In this context, the satellite-based parametric approach provides guidance on the phytoplankton bloom timing that contributes to determining the optimal timing of field measurements in DBO transects. The resulting experimental design of ship-based observations can maximize the complementary information obtained from field measurements along the DBO transects and satellite data. Extensive, sustained, and cost-effective observations by satellite contribute to addressing the detection and consistent monitoring of the biophysical responses to the changing environments in the Pacific Arctic.
